# Obituary

**DOI:** 10.1186/s13034-017-0199-7

**Published:** 2018-01-03

**Authors:** Joerg Fegert, Benedetto Vitiello, Janice Abbott

**Affiliations:** 1 Ulm, Germany; 2 Bethesda, USA; 3 Preston, UK

**Figure:**
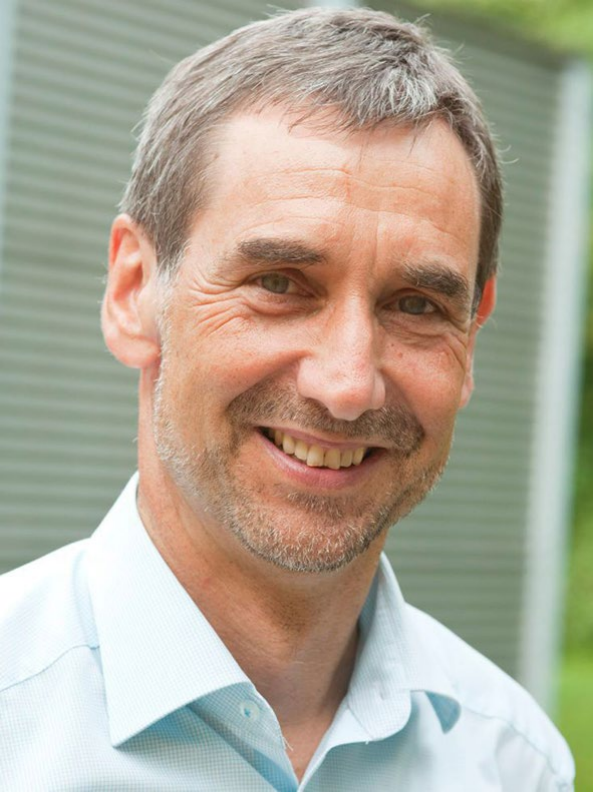
Prof. Dr. Lutz Goldbeck, * November the 25th 1958–^†^October 30th 2017

## Joerg Fegert and Benedetto Vitiello

Prof. Goldbeck passed away suddenly and unexpectedly due to a heart attack on October 30th, 2017. With him, CAPMH loses one of its original founders, and the entire field of mental health loses a most dedicated, energetic, and accomplished clinician, teacher, and researcher.

Prof. Goldbeck had a very intense and successful professional career, which was rich in accomplishments in a variety of areas. After graduating in Psychology at the University of Hamburg, Germany in 1984, he specialized in clinical psychology in 1988, and earned his Ph.D. (Dr. Phil.) in Psychology at the Freie Universität of Berlin, Institute of Philosophy, in 1993.

Following an initial clinical experience at the Department of Child and Adolescent Neurology and Psychiatry of the University Hospital in Berlin, he moved to the University of Ulm, where he worked as a pediatric psychologist in the Department of Pediatrics for 10 years. Building on his clinical work in consultation-liaison service, he started research projects on coping with chronic diseases, quality of life, and psychosocial interventions. He became a licensed psychotherapist for children, adolescents, and adults. In 2001, he joined the newly constituted Department of Child and Adolescent Psychiatry and Psychotherapy as its first principal clinical psychologist, where he also expanded the psychiatric outpatient clinic (Kinder- und Jugendpsychiatrische Institutsambulanz).

In 2004, Lutz Goldbeck completed the qualifications for the German academic habilitation on the quality of life of chronically ill children and adolescents, and received the *venia legendi* for child and adolescent psychotherapy and psychosomatics at the University of Ulm Medical School, becoming Associate Professor (APL-Professur) in 2007.

Since 2009, he was the head of the Section for Psychotherapy Research and Behavioral Medicine and head of the Centre for Training and Child and Adolescent Behavior Therapy at the Department of Child and Adolescent Psychiatry and Psychotherapy at the University of Ulm, where he became a full professor (W3) in 2014.

Prof. Goldbeck’s research focused on the psychosocial aspects of chronic pediatric conditions, coping, quality of life, psychiatric comorbidity, development and evaluation of psychosocial interventions, evaluation of mental health services, and interventions for children and adolescents with posttraumatic stress disorder. He initiated and was part of a number of international research collaborations, in both Europe and the United States. He was the recipient of a number of clinical and research awards, such as the Lilly Quality of Life Research Award and the Research Award of the German Heart Association for Children, in 2002. He was also awarded research and training grants from the German Research Foundation (DFG).

He was a co-founder of the journal Child and Adolescent Psychiatry and Mental Health (CAPMH) that received funding from the German Research Foundation DFG for establishing and implementing this first open access journal in the field of child and adolescent psychiatry and mental health. He served as deputy editor of CAPMH.

He served as a reviewer for numerous scientific journals and for different research organizations. He was member of the Society of Pediatric Psychology (American Psychological Association, Division 54), the German Psychological Society (DGPs), the International Society for the Prevention of Child Abuse and Neglect (ISPCAN) and of several associations and societies fighting against cystic fibrosis. He had become a member of the International Society of Traumatic Stress Studies (ISTSS) and was about to present at this year’s convention, when he suddenly passed away. He passed during a meeting of the board of the German-Speaking Society of Psychotraumatherapy (DeGPT), of which he was a board member. He had planned to organize the 2019 meeting of this society in Ulm.

Lutz was a very experienced supervisor of more than 50 academic medical and psychological dissertations. He trained numerous researchers and therapists. Lutz Goldbeck was a creative innovator in the field of collaborative research in child psycho-trauma therapy, including the application of web-based technologies for the delivery of therapeutic interventions in clinical settings. He founded the “Ulmer Online-Klinik” (Ulm Online-Clinic) and managed several projects and clinical trials on online psychotherapy. The recently founded transdisciplinary Centre for Trauma Research of the University of Ulm loses one of its most experienced and influential researchers.

Dr. Lutz Goldbeck leaves three children. In his spare time, he liked vacationing and sailing in Scandinavia, where he had many friends and acquaintances.

## Janice Abbott

The sudden death of Lutz Goldbeck at the age of 58 years has saddened the cystic fibrosis (CF) community. For more than two decades, Lutz devoted his time to the study of the psychosocial impact of CF disease. Lutz had a deep understanding of research methods that culminated in rigorously designed studies and exceptional research papers. He also had a desire to advance practice and improve the quality of life for children, young people and their families. Since the news of Lutz’s death has spread, I have received so many messages, citing examples of his contribution to the understanding and improvements in CF mental health care.

It is difficult to capture all of Lutz’s outstanding achievements in a short tribute. Here are a few highlights. In 2003, Lutz published his ‘Questions on Life Satisfaction for Adolescents and Adults with Cystic Fibrosis’ (FLZ-CF): a ‘quality of life’ instrument that provided meaningful data from young people with CF as to how the disease impacted their life. Systematically asking the patient ‘how they are’ was novel at this time and provided young people with a voice, and the scientific community with patient-reported outcome measure for CF. Using this instrument, Lutz undertook a series of cross sectional and longitudinal studies describing and evaluating the negative impact of CF disease on quality of life. More recently, Lutz directed a Cochrane Review (2014) that evaluated psychosocial and physical outcomes of psychological interventions in CF and highlighted the fact that more scientifically rigorous studies on psychological interventions were urgently needed. Indeed, in 2015, Lutz co-authored a wonderful web-based psychological support program for caregivers of children with cystic fibrosis which improved mental health and quality of life.

Lutz directed Germany’s contribution to the CF International Depression and Anxiety Epidemiological Study (TIDES, 2014). The results highlighted the magnitude of psychopathology among young people with CF and their caregivers. This led to the creation of the International Committee on Mental Health of which Lutz was a core member and in 2015 co-authored the CF Mental Health Guidelines that recommended annual mental health screening for young people with CF and their caregivers. To ensure the implementation of these guidelines, and evaluate their effectiveness across Europe, in 2016, the European Cystic Fibrosis Society formed the Mental Health Working Group of which Lutz was an active member. Lutz was very much a core part of the fabric of psychological research in CF. His sudden passing has left a ‘Lutz shaped hole’ and he will be terribly missed. Sincere condolences go to Christiane (we shared a wonderful time in Artimino, Italy, during the development of the CF Mental Health Guidelines) and his three sons of whom he was so proud.

